# Risk factors for acute kidney injury in pediatric intensive care units: a systematic review and meta-analysis

**DOI:** 10.1186/s12887-026-06555-6

**Published:** 2026-04-06

**Authors:** Rong Li, Qianqian Yang

**Affiliations:** Affiliated Hospital of Xuzhou Medical University, Xuzhou, 221000 China

**Keywords:** Acute kidney injury, Pediatric, Intensive care units, Risk factors, Meta-analysis

## Abstract

**Background:**

Acute kidney injury (AKI) is one of the common and severe complications in pediatric intensive care units (PICUs), closely associated with increased mortality, prolonged hospital stays, and heightened healthcare burdens among pediatric patients. A meta-analysis examined the associated risk factors for AKI in PICUs patients.

**Methods:**

A systematic search was conducted in PubMed, Embase, Web of Science, and the Cochrane Library databases from their inception to August 20, 2025, to identify observational studies reporting risk factors for AKI in pediatric PICU patients. Two researchers independently performed literature screening, data extraction, and quality assessment. Stata 15 software was used to calculate pooled odds ratios (ORs) with 95% confidence intervals (CIs), assess heterogeneity, and analyze publication bias.

**Results:**

Thirteen articles involving 8557 patients were included, meta-analysis results suggested that age (per year increase) [OR = 1.64, 95%CI (1.03, 2.61)], female [OR = 1.45, 95%CI (1.11, 1.91)], sepsis [OR = 5.98, 95%CI (3.10, 11.51)], MODS [OR = 3.92, 95%CI (2.29, 6.71)], coagulopathy [OR = 2.24, 95%CI (1.56, 3.24)], nephrotoxic drugs [OR = 2.87, 95%CI (2.16, 3.80)], mechanical ventilation [OR = 2.39, 95%CI (1.28, 4.47)]] were associated with the occurrence of AKI in PICUs.

**Conclusion:**

The Meta-analysis results of this study revealed several important AKI risk factors, including age, gender, sepsis, multiple organ dysfunction, coagulation disorders, nephrotoxic drug use and mechanical ventilation.

**Supplementary Information:**

The online version contains supplementary material available at 10.1186/s12887-026-06555-6.

## Background

Acute kidney injury (AKI) is a syndrome characterized by a rapid decline in renal function, typically marked by a significant increase in serum creatinine levels and/or a reduction in urine output [1]. With advancements in critical care and pediatric medicine, the significance of AKI in pediatric intensive care units (PICUs) has grown increasingly evident [2]. Current studies show that the incidence of AKI in PICU patients ranges from 20% to 40%, with certain high-risk groups exceeding 50%. Once AKI occurs, it not only increases short-term mortality but also significantly prolongs hospital stays, escalates medical costs, and may result in long-term renal insufficiency or progress to chronic kidney disease (CKD) [3, 4]. This can severely impact children’s growth and development, as well as their long-term quality of life. International multicenter data further reveal that AKI increases the risk of in-hospital mortality by 3 to 5 times [5]. Even among survivors discharged from the hospital, long-term complications such as hypertension, proteinuria, and cardiovascular events persist.

There are significant differences between children and adults in the epidemiology, pathophysiology, and distribution of risk factors for AKI [6]. Pediatric kidneys have an immature structure and function, with lower glomerular filtration rates (GFR), reduced renal blood flow, and insufficient tubular concentrating capacity compared to adults. This renders pediatric kidneys more sensitive to ischemia-hypoxia, hemodynamic fluctuations, and nephrotoxic medications [7]. Concurrently, pediatric ICU patients present with complex underlying conditions, commonly involving severe infections, sepsis, shock, respiratory failure, and multiple organ dysfunction. These factors can directly or indirectly damage the kidneys, increasing the risk of AKI. Recent advancements in critical care medicine have introduced new challenges [8]. The extensive use of mechanical ventilation, extracorporeal membrane oxygenation (ECMO), vasoactive agents, broad-spectrum antibiotics, antifungal medications, and chemotherapeutic drugs—while saving critically ill children’s lives—increases exposure to nephrotoxic agents and the likelihood of AKI [9]. Additionally, some pediatric patients present with congenital renal developmental anomalies, post-operative status following complex congenital heart disease surgery, or pre-existing chronic conditions [10]. These uncontrollable congenital factors also heighten susceptibility to AKI.

From a global perspective, AKI is widely recognized as a crucial prognostic factor in critically ill pediatric patients. The 2012 kidney disease: Improving Global Outcomes (KDIGO) guidelines established the first standardized diagnostic criteria for AKI severity and emphasized the importance of early identification and intervention [11, 12]. The latest 2021 global epidemiological study on AKI indicates that despite improving diagnostic and treatment systems for adult AKI, research and intervention for pediatric AKI remain inadequate [13]. Particularly in low- and middle-income countries, AKI-related mortality rates remain as high as 30%–40% due to resource scarcity, delayed diagnosis, and lack of standardized treatment. In contrast, high-income countries exhibit higher rates of AKI identification and intervention, leading to relatively improved outcomes [14]. However, evidence-based understanding of risk factors in pediatric populations remains inadequate, representing a significant research gap. This underscores that identifying and intervening in high-risk factors for pediatric AKI is a critical focus in current global kidney protection research [15, 16].

Existing literature suggests that the development of AKI in children in the PICU results from a multifactorial process involving complex interactions between various risk factors. Sepsis is widely recognized as a major risk factor, and its mechanism involves microcirculatory disorders caused by inflammatory storms, altered vascular permeability, redistribution of renal blood flow, and direct toxicity of inflammatory mediators to the renal tubules [17]. Hypotension and shock lead to inadequate renal perfusion, exacerbate ischemia and hypoxia, and promote renal cell apoptosis and necrosis [18]. However, the results of current studies on risk factors for AKI in children remain highly variable and controversial. On the one hand, most of the studies are single-center retrospective cohorts with limited sample sizes and large heterogeneity in study design and inclusion populations; on the other hand, the diagnostic criteria for AKI are not standardized, which leads to significant differences in the incidence of AKI and identification of risk factors.

In this study, a meta-analysis was conducted to comprehensively search and strictly screen relevant observational studies both domestically and internationally, integrate the existing evidence-based findings, quantitatively assess the correlation between multiple potential risk factors and the occurrence of AKI in children within the PICU, improve both short-term survival and long-term prognosis of critically ill infants, and provide a scientific basis for the construction of effective prevention and treatment strategies for AKI in the pediatric population.

## Methods

This systematic review and meta-analysis will adhere rigorously to the PRISMA (Preferred Reporting Items for Systematic Reviews and Meta-Analyses) guidelines [19]. Additionally, it has been registered in the Prospero database under registration number CRD420251125712.

### Inclusion and exclusion criteria

This study included observational studies (cohort, case-control, and cross-sectional) involving children aged 18 years or younger who were hospitalized in pediatric intensive care units (PICU), including those in pediatric cardiac ICUs. Studies were required to explicitly use KDIGO-recognized criteria, pRIFLE criteria, or serum creatinine for diagnosing acute kidney injury (AKI) and report the association between AKI and at least one potential risk factor. Additionally, studies should provide effect size data such as odds ratios (OR) and their corresponding 95% confidence intervals (CI), and these data should be extractable or convertible. Only studies with complete and usable data, available in any language and published at any time, were included. Full-text availability was also a requirement.

We excluded non-original studies, such as reviews, meta-analyses, case reports, conference abstracts, editorials, and expert opinions. Studies that did not focus on children in PICUs or that included adults and neonates were also excluded. Additionally, studies without clear diagnostic criteria for AKI or those not following internationally recognized guidelines were excluded. We excluded studies that did not report risk factors associated with AKI or those for which quantitative data (such as ORs) could not be extracted, and for which the required data could not be obtained by contacting the authors. Duplicate publications or analyses were excluded, retaining only the study with the largest sample size and most complete data.

### Literature retrieval

Two independent researchers conducted systematic searches across the following databases: PubMed, Web of Science, Embase, and the Cochrane Library. The search covered all records up to August 20, 2025. The search strategy combined Medical Subject Headings (MeSH) terms with free-text keywords focusing on four key components: “acute kidney injury,” “pediatric,” “intensive care units,” and “risk factor.” The full search strategy is outlined in Supplementary Material Table S1. Adjustments were made to the search strategy for each database. To ensure thoroughness, the reference lists of included studies were manually searched for any relevant publications that might have been missed. Any disagreements in the search or screening process were resolved by consulting a third researcher.

### Study selection

The study selection process was carried out by two researchers using EndNote 21 software. The initial screening involved reviewing titles and abstracts to exclude studies that clearly did not meet the inclusion criteria. Remaining studies underwent full-text review for eligibility based on the inclusion and exclusion criteria. Any disagreements were resolved through discussion, and if consensus could not be reached, a third researcher made the final decision to ensure objectivity and consistency in the screening process.

### Data extractions

Two researchers independently extracted relevant data from eligible studies using an Excel sheet. Extracted data included basic study information (first author, year of publication, country, and study design) and characteristics of the study population (sample size, number of AKI cases, gender distribution, mean age, and diagnostic criteria for AKI). Disagreements during data extraction were resolved through discussion, with a third investigator making the final decision if necessary. Although inter-rater reliability was not formally assessed, future analyses could benefit from evaluating consistency between reviewers using Kappa statistics to strengthen the reliability of the findings.

### Quality evaluation

The risk of bias in the selected studies was assessed independently by two reviewers, with a cross-check of their findings. For cohort and case-control studies, we applied the Newcastle Ottawa Scale (NOS) [20] to evaluate study quality. This scale assesses three key areas: selection of the population, comparability, and the exposure or outcome measures, with a total of eight items yielding a maximum of nine points. Studies scoring between 0 and 4 were considered of low quality and excluded. For cross-sectional studies, the AHRQ [21] quality assessment tool was used, which evaluates the study’s design rationale, the representativeness of the sample, clarity in defining exposure and outcomes, the accuracy of data collection, the appropriateness of statistical methods, and the completeness of reporting.

### Statistical analysis

This study used Stata 15 software to pool the corresponding 95% confidence intervals (CI) and odds ratios (OR) from each included study. Given the variability expected across studies in terms of design, populations, and contexts, the random-effects model was deemed appropriate for all analyses. In this study, we summarized effect measures reported in different studies, including relative risk (RR) and odds ratio (OR). To present the results uniformly, all effect sizes were converted to odds ratios (OR). For studies that reported RR, we used the following formula to convert it to OR.

$$\mathrm{OR}=\mathrm{RR}\left(1-\mathrm{Pcontrol}+\mathrm{Pcontrol}\times\mathrm{RR}\right)$$

where Pcontrol is the event rate in the control group. To assess heterogeneity, we employed the I² statistic, with an I² value exceeding 50% considered indicative of substantial heterogeneity. If significant heterogeneity was found, sensitivity analyses were conducted to explore potential factors influencing the pooled effect size. Specifically, we assessed how the effect estimates changed when individual studies were removed, which is critical for interpreting the robustness and consistency of the results. Publication bias was assessed using funnel plot asymmetry and Egger’s test. A *p*-value < 0.05 indicated publication bias, while a *p*-value > 0.05 suggested no bias.

## Results

### Literature screening results

**Fig. 1:**
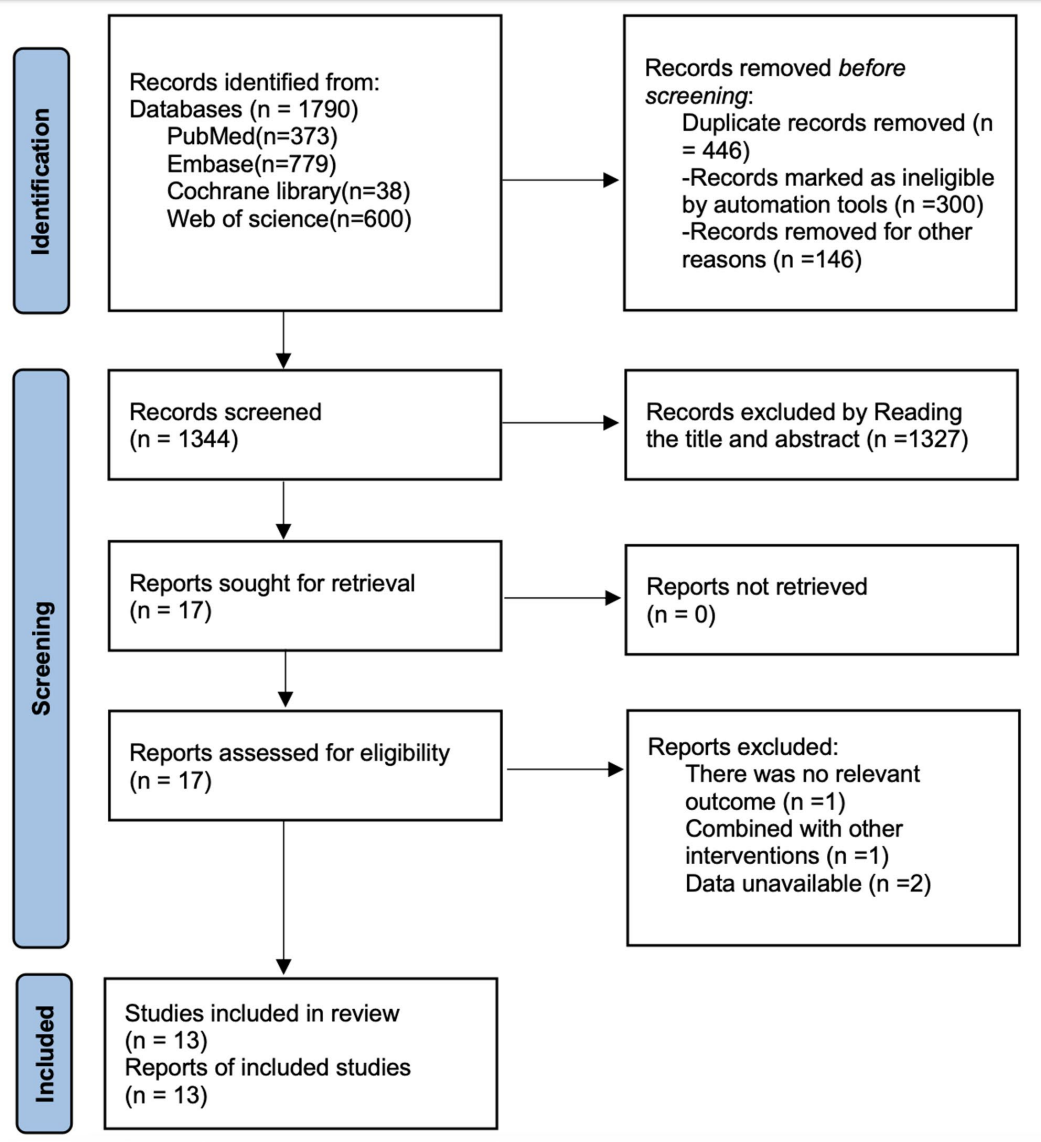
Literature search flow chart

As shown in Fig. 1, a total of 1790 documents were obtained by searching PubMed (*n* = 373), Embase (*n* = 779), Cochrane library (*n* = 38), and Web of science (*n* = 600), and a total of 446 duplicates were removed by removing 446 duplicates, 1327 articles were removed by reading the titles and abstracts, and 4 were removed by reading the full text. articles, and finally 13 studies [22–34] were included.

### Basic characteristics of the included studies

Thirteen articles involving 8557 patients were included in this study, out of which one study was a case-control study [31], one was a cross-sectional study [28], and eleven studies were cohort studies, with a total number of AKIs of 1986 patients with an age range of 1.3-9 years old, and the specific basic characteristics are shown in Table 1. The level of agreement was medium according to the interpretation criteria for Kappa values [35], the results of this analysis suggest a degree of consistency in the review process.

**Table 1:** Basic characteristics of the included studies

study	year	country	study design	sample size	No of AKI	gender(M/F)	mean age(years)	Diagnosis of AKI	Regression model
Bailey	2007	Canada	cohort study	985	44	536/449	6	serum creatinine	multivariate Logistic regression analysis
Celegen	2023	Turkey	cohort study	360	63	185/175	5.5	KDIGO criteria	multivariate Logistic regression analysis
Chalisah	2024	Indonesia	cohort study	255	68	134/121	5.8	pRIFLE criteria	multivariate Logistic regression analysis
de Souza	2023	Brazil	cohort study	108	47	65/43	9	KDIGO criteria	multivariate Logistic regression analysis
De	2020	Italy	cohort study	811	222	464/347	5.5	KDIGO criteria	multivariate Logistic regression analysis
Duan	2019	China	cohort study	127	55	61/66	1.3	KDIGO criteria	multivariate Logistic regression analysis
Esmaeili	2024	Iran	cross‐sectional	253	58	139/114	1.75	KDIGO criteria	multivariate Logistic regression analysis
Gupta	2016	India	cohort study	536	230	286/250	4.5	pRIFLE criteria	multivariate Logistic regression analysis
Hui	2023	China	cohort study	254	106	148/106	4.9	KDIGO criteria	multivariate Logistic regression analysis
Keneni	2023	Ethiopia	case-control	241	81	121/120	5.8	KDIGO criteria	multivariate Logistic regression analysis
Rustagi	2017	India	cohort study	380	53	194/186	6.4	pRIFLE criteria	multivariate Logistic regression analysis
Serna	2017	Germany	cohort study	382	44	202/180	6.45	KDIGO criteria	multivariate Logistic regression analysis
Slater	2016	Canada	cohort study	3865	915	2164/1701	3.4	KDIGO criteria	multivariate Logistic regression analysis

### Risk of bias results

The quality assessment of this study is detailed in Table 2. One cross-sectional study was rated as moderate quality, while one study in the cohort study was scored 7 points, four articles were scored 8 points, and six articles were scored 9 points, one case control was scored 9 points, indicating a high overall quality.

**Table 2:** Newcastle-Ottawa Scale (NOS) score results

cross-sectional											
Study	Whether the source of the information is clear	Whether exposed and non-exposed groups are listed	Whether a time was given to identify patients	If not, population derived, whether the subjects were consecutive	Whether the subjective factors of the evaluator cover up other aspects of the research object	Any assessment performed to ensure quality is described	The rationale for excluding any patients from the analysis was explained	Describe measures to evaluate and/or control for confounding factors	explain how missing data were handled in the analysis	Response rates and the completeness of data collection are summarized	If there is follow-up, identify the percentage of patients with expected incomplete data or follow-up results
Esmaeili 2024 [28]	Yes	Unclear	Yes	Yes	Yes	Yes	Yes	Yes	Yes	Unclear	Yes
cohort study											
Study	Representativeness of the exposed group	Selection of non-exposed groups	Determination of exposure factors	Identification of outcome indicators not yet to be observed at study entry	Comparability of exposed and unexposed groups considered in design and statistical analysis	design and statistical analysis	Adequacy of the study's evaluation of the outcome	Adequacy of follow-up in exposed and unexposed groups	Total scores		
Bailey 2007 [22]	*	*	*	*	**	*	*	*	9		
Celegen 2023 [23]	*	*	*	/	**	*	*	*	8		
Chalisah 2024 [24]	*	*	*	/	**	*	*	*	8		
de Souza 2023 [25]	*	*	*	*	**	*	*	*	9		
De 2020 [26]	*	*	*	*	**	*	*	*	9		
Duan 2019 [27]	*	*	*	*	**	*	*	*	9		
Serna 2017 [33]	*	*	*	/	**	/	*	*	7		
Gupta 2016 [29]	*	*	*	*	**	*	*	*	9		
Hui 2023 [30]	*	*	*	/	**	*	*	*	8		
Slater 2016 [34]	*	*	*	/	**	*	*	*	8		
Rustagi 2017 [32]	*	*	*	*	**	*	*	*	9		
case control											
Study	Is the case definition adequate?	Representativeness of the cases	Determination of control group	Definition of Controls	Comparability of cases and controls based on the design or analysis	Ascertainment of exposure	Same method of ascertainment for cases and controls	Non response	Total scores		
Keneni 2023 [31]	*	*	*	*	**	*	*	*	9		

* means one scores

** means two scores

### Meta-analysis results

#### Age (per year increase)

**Fig. 2:**
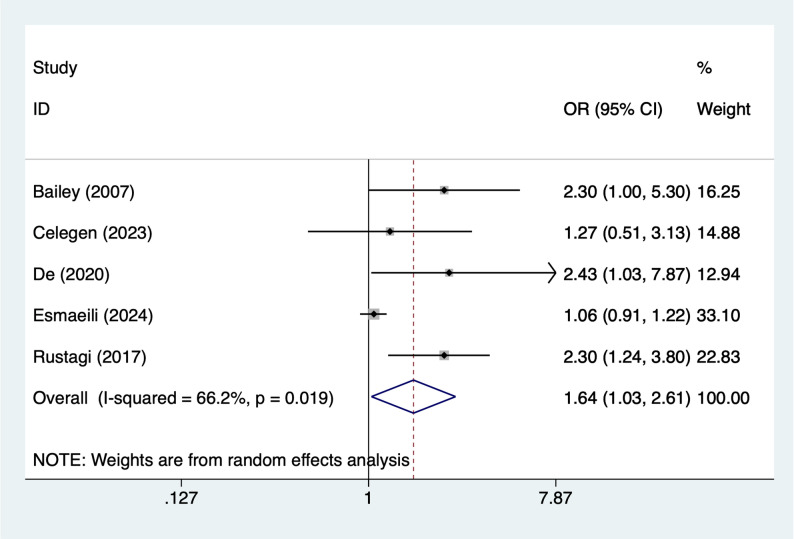
Forest plot of meta-analysis for younger age

Five articles mentioned age (per year increase), heterogeneity test (I^2^ = 76.3%, *P* = 0.001), and random effects model were used for the analysis, and the results of the analysis (Fig. 2) suggested that age (per year increase) [OR = 1.64, 95%CI (1.03, 2.61)] was associated with the occurrence of AKI in PICUs. Due to the large heterogeneity, sensitivity analyses were performed using literature-by-exclusion, and the results of the analyses (Supplementary Material Fig. S1) suggested that the findings were not influenced by a single study.

#### Female

**Fig. 3:**
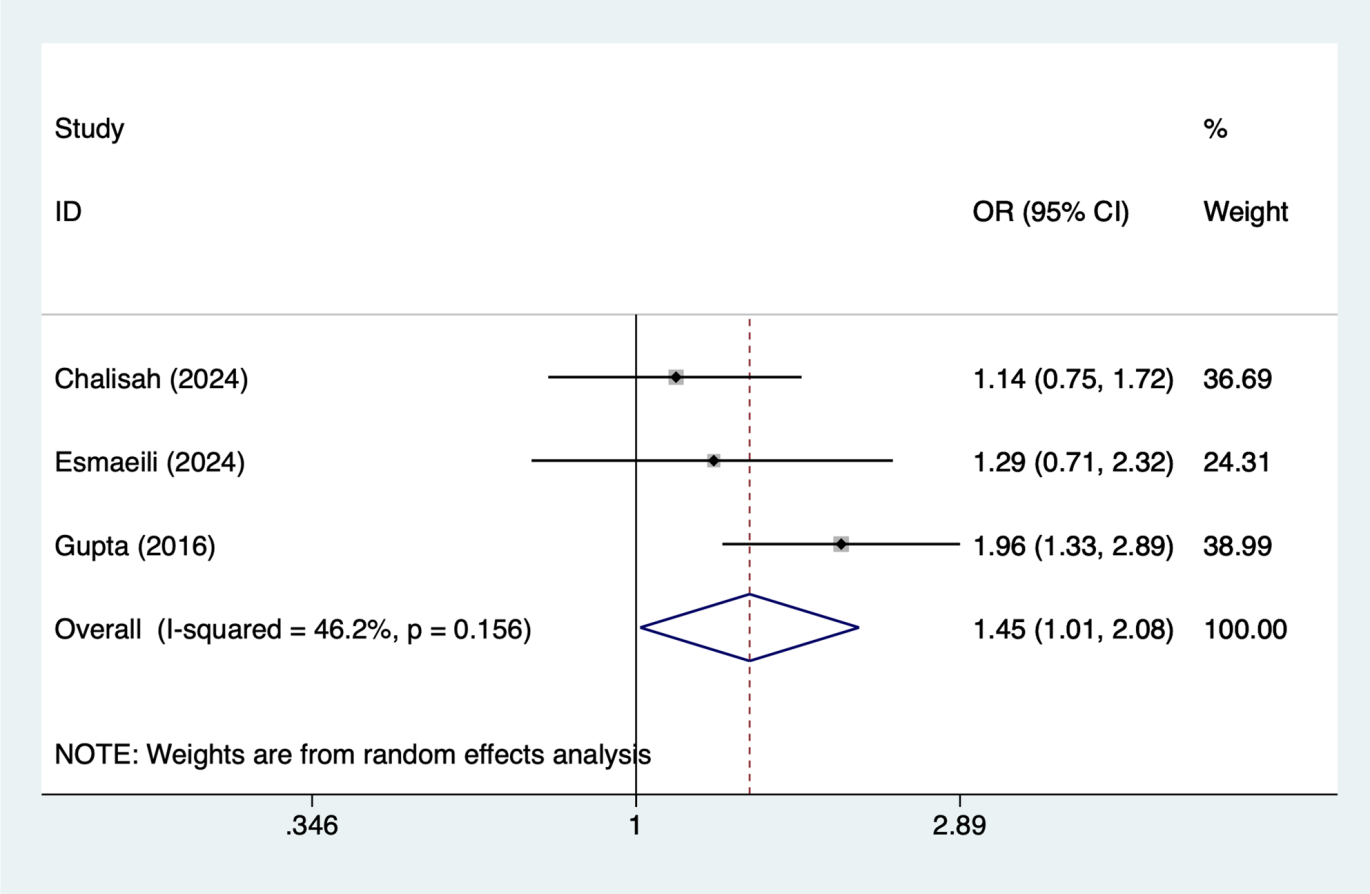
Forest plot of meta-analysis for female gender

Three articles mentioned female, heterogeneity test (I^2^ = 46.2%, *P* = 0.156), and random effects model were used for the analysis, and the results of the analysis (Fig. 3) suggested that female [OR = 1.45, 95%CI (1.01, 2.08)] was associated with the occurrence of AKI in PICUs.

#### Sepsis

**Fig. 4:**
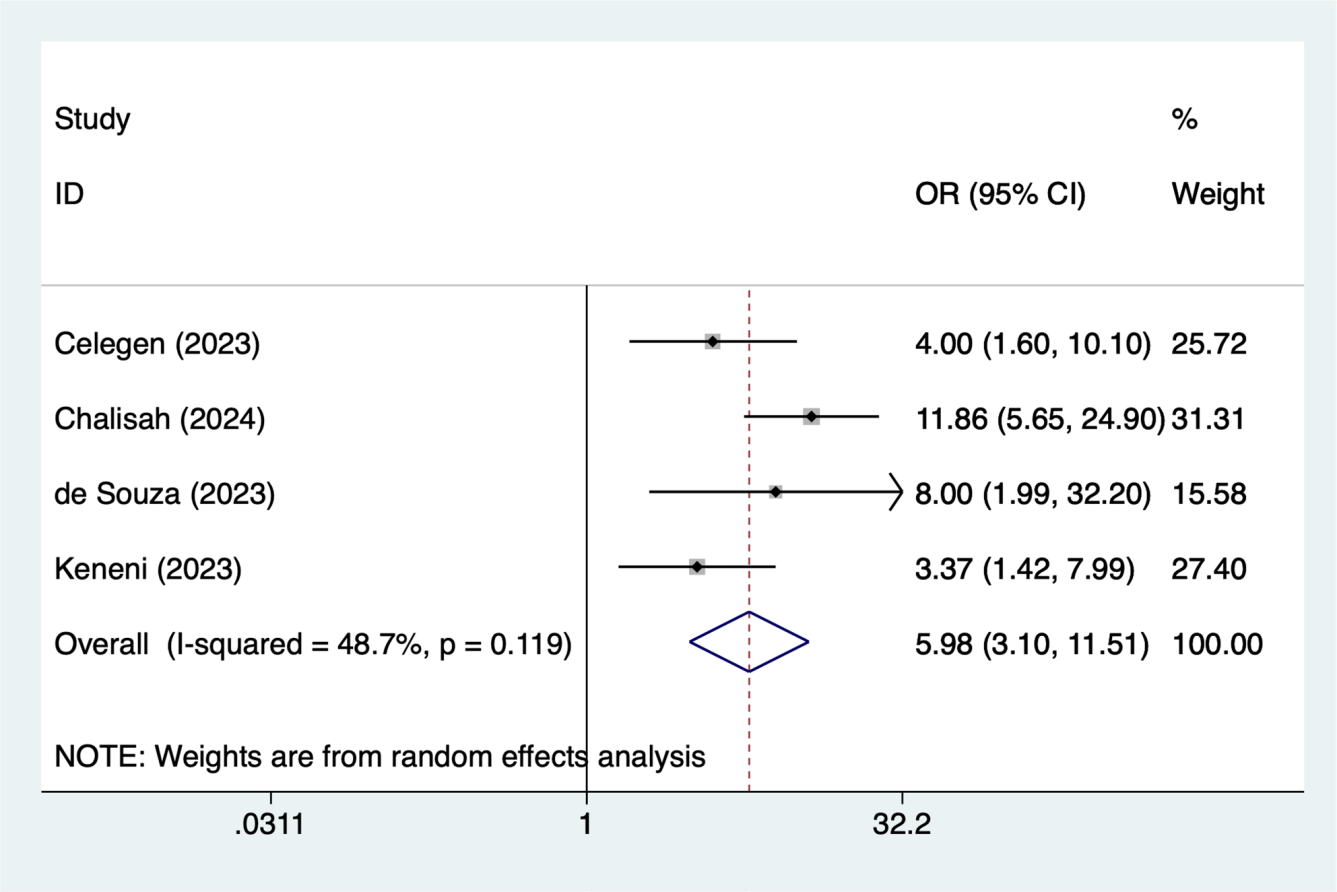
Forest plot of meta-analysis for sepsis

Four articles mentioned sepsis, heterogeneity test (I^2^ = 48.7%, *P* = 0.119), and random effects model were used for the analysis, and the results of the analysis (Fig. 4) suggested that sepsis [OR = 5.98, 95%CI (3.10, 11.51)] was associated with the occurrence of AKI in PICUs.

### Multiple organ dysfunction syndrome

**Fig. 5:**
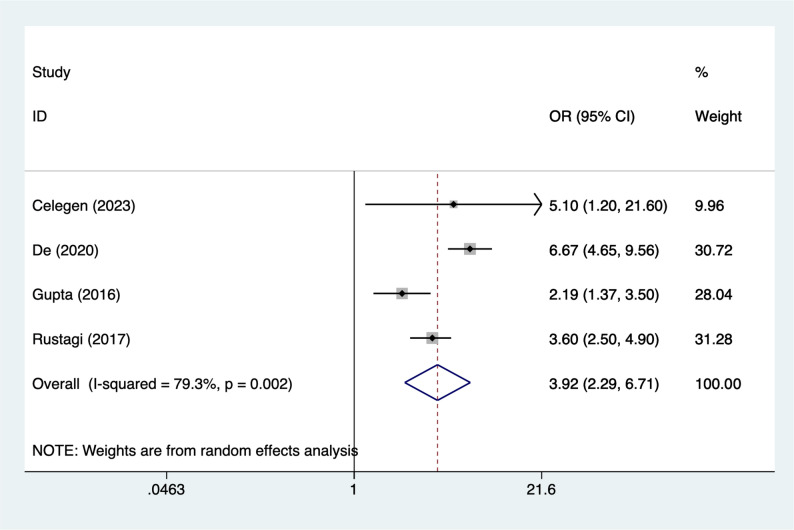
Forest plot of meta-analysis for Multiple Organ Dysfunction Syndrome (MODS)

Four articles mentioned multiple organ dysfunction syndrome (MODS), heterogeneity test (I^2^ = 79.3%, *P* = 0.002), and random effects model were used for the analysis, and the results of the analysis (Fig. 5) suggested that MODS [OR = 3.92, 95%CI (2.29, 6.71)] was associated with the occurrence of AKI in PICUs. Due to the large heterogeneity, sensitivity analyses were performed using literature-by-exclusion, and the results of the analyses (Supplementary Material Fig. S2) suggested that the findings were not influenced by a single study.

### Coagulopathy

**Fig. 6:**
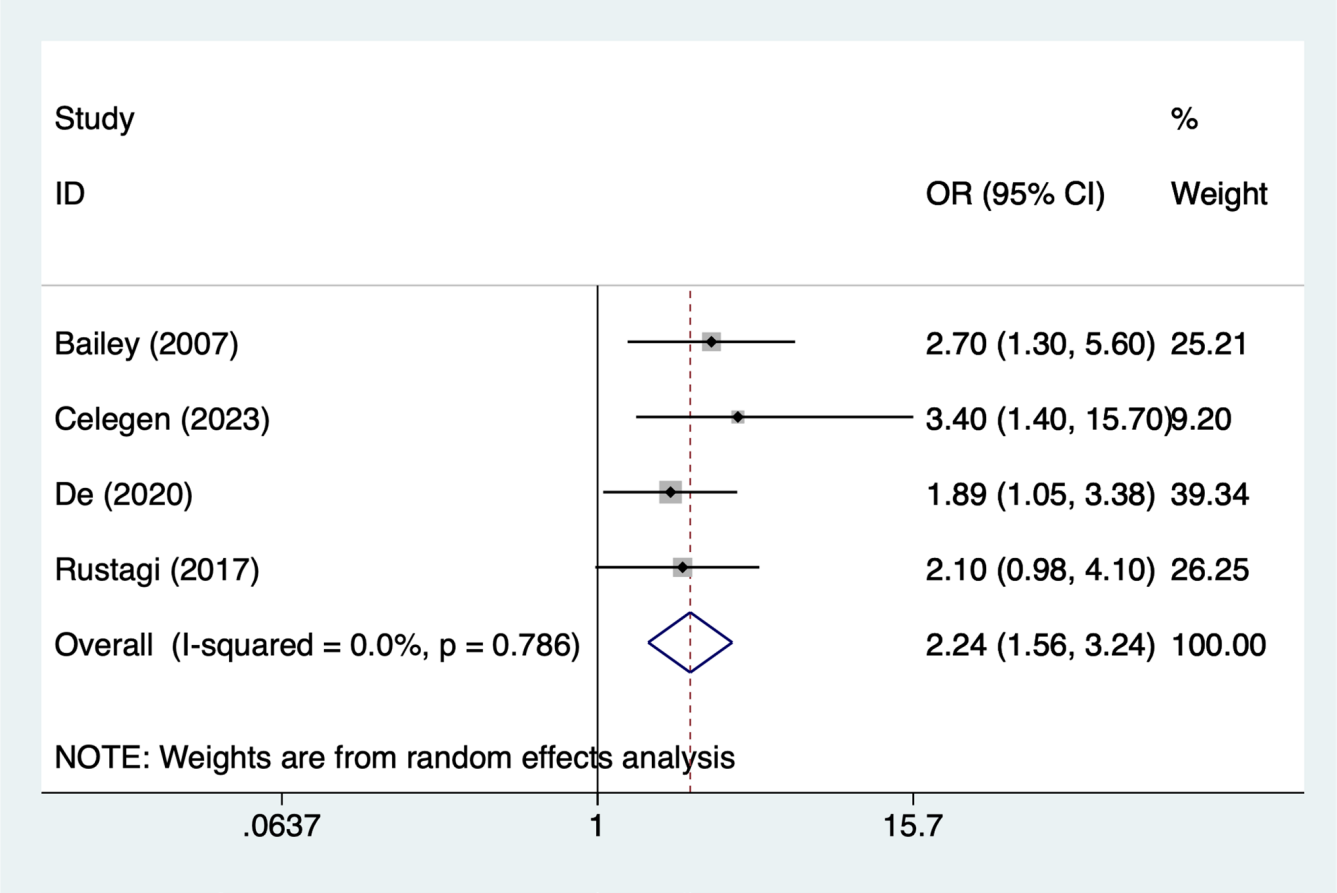
Forest plot of meta-analysis for coagulopathy

Four articles mentioned coagulopathy, heterogeneity test (I^2^ = 0%, *P* = 0.786), and random effects model were used for the analysis, and the results of the analysis (Fig. 6) suggested that coagulopathy [OR = 2.24, 95%CI (1.56, 3.24)] was associated with the occurrence of AKI in PICUs.

### Nephrotoxic drugs

**Fig. 7:**
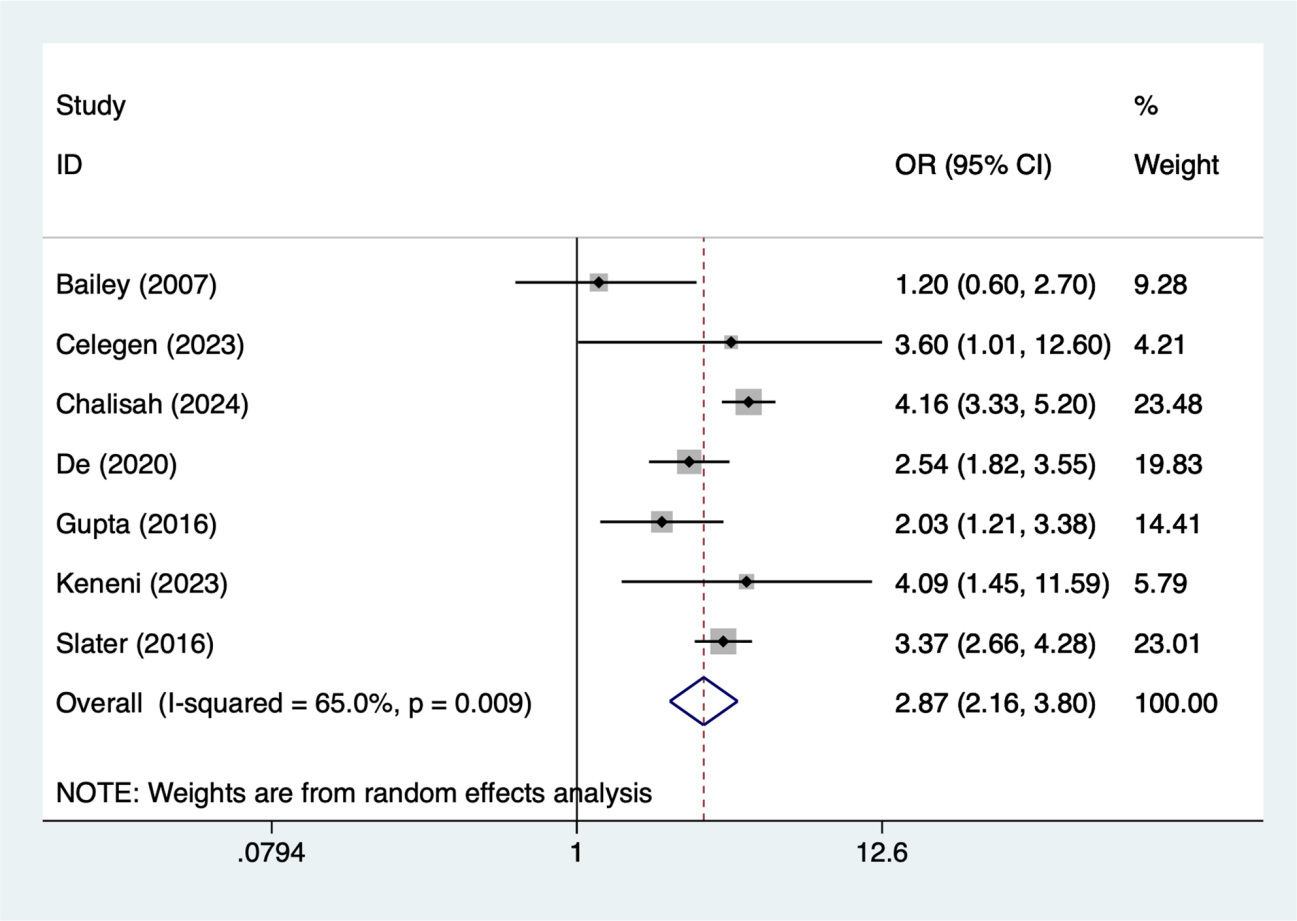
Forest plot of meta-analysis for nephrotoxic drugs

Seven articles mentioned nephrotoxic drugs (aminoglycosides, vancomycin, Nonsteroidal Anti-Inflammatory Drugs), heterogeneity test (I^2^ = 65%, *P* = 0.009), and random effects model were used for the analysis, and the results of the analysis (Fig. 7) suggested that nephrotoxic drugs [OR = 2.87, 95%CI (2.16, 3.80)] was associated with the occurrence of AKI in PICUs. Due to the large heterogeneity, sensitivity analyses were performed using literature-by-exclusion, and the results of the analyses (Supplementary Material Fig. S3) suggested that the findings were not influenced by a single study.

### Mechanical ventilation

**Fig. 8:**
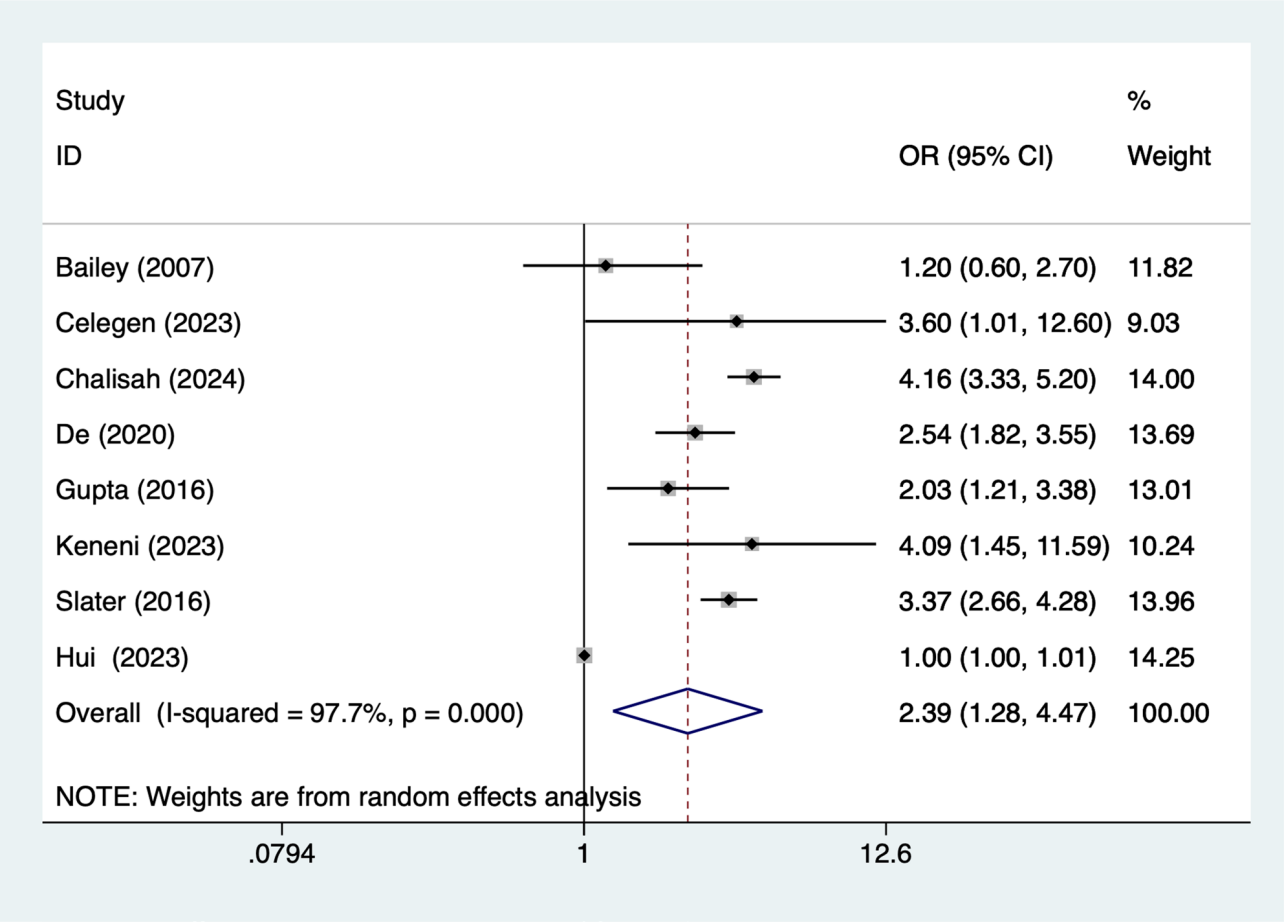
Forest plot of meta-analysis for mechanical ventilation

Nine articles mentioned mechanical ventilation, heterogeneity test (I^2^ = 97.7%, *P* = 0.001), and random effects model were used for the analysis, and the results of the analysis (Fig. 8) suggested that mechanical ventilation [OR = 2.39, 95%CI (1.28, 4.47)] was associated with the occurrence of AKI in PICUs. Due to the large heterogeneity, sensitivity analyses were performed using literature-by-exclusion, and the results of the analyses (Supplementary Material Fig. S4) suggested that the findings were not influenced by a single study.

### Publication bias

The current study detected its publication bias by funnel plot and egger test. The funnel plot results (Supplementary material Fig. S5-fig. S11) suggested that young age (Egger *P* = 0.002), mechanical ventilation (*P* = 0.047), nephrotoxic drugs (*P* = 0.023) funnel plots were asymmetric, and the *P*-value of Egger was less than 0.05, which proved that there was publication bias. These results were therefore further analyzed by the trim-and-fill method, and the trim-and-fill results (Supplementary Material Fig. S12- Fig. S14) suggested that even the presence of publication bias did not affect the results. None of the remaining outcomes had publication bias.

## Discussion

This study systematically evaluated and performed a meta-analysis on the major risk factors for AKI in PICUs. The results revealed several key risk factors significantly associated with the occurrence of AKI, including age (per year increase), female gender, sepsis, MODS, coagulation disorders, nephrotoxic drugs, and mechanical ventilation. These findings provide valuable insights for clinical practice, supporting early screening, risk assessment, and intervention strategies to mitigate AKI in PICU patients.

The meta-analysis highlighted that age (per year increase) significantly increased the risk of AKI. Older pediatric patients may be more susceptible to acute kidney injury due to physiological changes in the kidneys, comorbidities, and greater likelihood of receiving nephrotoxic therapies. This underscores the importance of considering age as a critical factor when assessing the risk of AKI in critically ill children. This study also found that women were at higher risk of developing AKI than men, this gender difference may be related to physiological and endocrine differences, and female patients may be more susceptible in the context of infections, drug reactions, or other diseases [36]. Although this finding requires further validation, it provides clinicians with an important reference for considering gender when assessing AKI risk. Particularly in terms of treatment strategies, female patients may require more refined interventions, such as tailored dosages or specific monitoring due to their unique risk factors and responses to treatment [37]. Sepsis, as a common and serious complication, is one of the important risk factors for AKI in the PICU. The systemic inflammatory response, immune system disorders, and hemodynamic changes caused by sepsis may lead to acute kidney injury by affecting renal perfusion and tubular function [38]. Therefore, clinicians should be highly vigilant for the occurrence of AKI in children with sepsis and take active monitoring and intervention measures, such as early recognition, rational use of antibiotics and rehydration management. The results of Meta-analysis showed that MODS significantly increased the risk of AKI.MODS usually occurs in critically ill children and involves the functional failure of multiple organs, which is a manifestation of high clinical morbidity and mortality [39]. MODS may lead to impaired renal function through direct or indirect effects on the kidneys [40]. Therefore, clinicians must pay close attention to renal function when managing patients with MODS and provide timely renoprotective therapy and multi-organ support to reduce the incidence of AKI. Meta-analyses have shown that coagulation dysfunction is strongly associated with the development of AKI. Coagulation dysfunction may contribute to the development of AKI by triggering microvascular thrombosis or increasing the risk of inadequate renal perfusion [41]. Clinically, children with coagulation abnormalities require enhanced renal monitoring, especially during surgery or critical care, and prompt measures to correct coagulation to reduce the risk of AKI. Meta-analyses have shown that children on nephrotoxic drugs have a greatly increased risk of AKI. Such drugs include antibiotics, chemotherapeutic agents and other drugs commonly used in clinical practice, which can cause renal injury through direct toxic effects or indirect hemodynamic alterations [42]. Therefore, clinicians should carefully monitor the renal function of children using nephrotoxic drugs, adjust the drug regimen in time, and avoid overuse or overdose of drugs [43]. The results of Meta-analysis showed that mechanical ventilation was closely associated with the development of AKI. Mechanical ventilation may increase the risk of AKI through mechanisms that affect blood circulation, lung function, oxygenation status, and respiratory-induced systemic inflammatory responses [44]. In clinical practice, for children requiring mechanical ventilation, especially those with severe conditions, in addition to routine ventilation support, the monitoring of renal function should be strengthened, and individualized treatment plans should be formulated to avoid the occurrence of AKI [45].

In the present study, several factors (younger age, sepsis, MODS, nephrotoxic drugs, mechanical ventilation) showed a high degree of heterogeneity, which may be related to differences in different study designs, sample sources and assessment methods. To reduce the interference of these factors on the results, sensitivity analyses were performed in this study, which showed that most of the findings were not affected by a single study, suggesting a high degree of robustness. However, the presence of heterogeneity still suggests that future studies should further standardize the study design and minimize potential biasing factors to improve the accuracy and generalizability of the results. In the analysis of publication bias, this study found publication bias in studies related to younger age, mechanical ventilation and nephrotoxic drugs. Nevertheless, after further analyses using the trim-and-fill method, the results showed that publication bias did not significantly affect the conclusions of this study. The existence of publication bias may be related to factors such as study publication tendency, financial support, and the significance of study results. Therefore, future studies should enhance the transparency of data and the quality of reporting to ensure that the results of all relevant studies are fully and fairly presented.

### Strengths and limitations

The strength of this study lies in its large study sample and strict inclusion criteria, which covered a wide range of potential AKI risk factors, which were systematically evaluated and Meta-analysis. In addition, this study used a high-quality risk of bias assessment tool to ensure the reliability of the results.

However, t This study has several limitations. First, while the included studies used a variety of designs (including case-control, cross-sectional, and cohort studies), there was a high degree of heterogeneity among them. This variability in study design, sample sources, and assessment methods may have affected the stability and generalizability of the results. Second, some studies did not provide detailed information on certain potential risk factors, such as drug use and etiology, which limited our ability to explore these factors more comprehensively. Finally, most of the included studies were single-center studies, and there was a lack of data from large-scale, multicenter studies, which may have introduced regional bias and limited the broader applicability of the findings. Additionally, the consistency between reviewers’ assessments was not evaluated, which could introduce uncertainty regarding the robustness of the review process. To strengthen the reliability of future analyses, it would be beneficial to assess inter-rater agreement using Kappa statistics to evaluate the consistency between reviewers and minimize potential bias in the review process.

## Conclusion

This meta-analysis identified several significant risk factors for AKI in PICU patients, including age, gender, sepsis, MODS, coagulation disorders, nephrotoxic drug use, and mechanical ventilation. These findings provide valuable clinical insights for the early identification, risk assessment, and intervention of AKI in the PICU setting. Despite these important contributions, the study also highlighted the challenges of heterogeneity and publication bias, suggesting that future research should focus on multi-center studies with larger sample sizes and explore additional AKI risk factors to further refine clinical management strategies.

## Supplementary Information

Supplementary Material 1.

## Data Availability

The data used for the analyses are available upon reasonable request from the first author. The data sets supporting the conclusions of this article are included within the article and its additional files.

## Abbreviations

AKI: Acute kidney injury
ORs: Odds ratios
CIs: Confidence intervals
PICUs: Pediatric intensive care units
CKD: Chronic kidney disease
GFR: Glomerular filtration rates
ECMO: Extracorporeal membrane oxygenation
