# Supplementary material for: Risk factors for acute kidney injury in pediatric intensive care units: a systematic review and meta-analysis
Source: BMC Pediatr. 2026 Apr 6;26:440. doi: 10.1186/s12887-026-06555-6 (PMC13188678; doi:10.1186/s12887-026-06555-6)
Supplement: Supplementary file 1 — Supplementary Material 1. [file 12887_2026_6555_MOESM1_ESM.docx]

Table S1 search strategy

(((("Child"[Mesh]) OR ((((Child[Title/Abstract]) OR (Children[Title/Abstract])) OR (Pediatrics[Title/Abstract])) OR (Pediatric[Title/Abstract]))) AND (("Intensive Care Units"[Mesh]) OR ((((((((((Intensive Care Units[Title/Abstract]) OR (Intensive Care Unit[Title/Abstract])) OR (Unit, Intensive Care[Title/Abstract])) OR (ICU Intensive Care Units[Title/Abstract])) OR (ICU, Pediatric[Title/Abstract])) OR (ICUs, Pediatric[Title/Abstract])) OR (Pediatric ICUs[Title/Abstract])) OR (Pediatric ICU[Title/Abstract])) OR (Pediatric Intensive Care Unit[Title/Abstract])) OR (Pediatric Intensive Care Units[Title/Abstract])))) AND (("Acute Kidney Injury"[Mesh]) OR (((((((((((((((((((((((((Acute Kidney Injury[Title/Abstract]) OR (Acute Kidney Injuries[Title/Abstract])) OR (Kidney Injuries, Acute[Title/Abstract])) OR (Kidney Injury, Acute[Title/Abstract])) OR (Acute Renal Injury[Title/Abstract])) OR (Acute Renal Injuries[Title/Abstract])) OR (Renal Injuries, Acute[Title/Abstract])) OR (Renal Injury, Acute[Title/Abstract])) OR (Kidney Failure, Acute[Title/Abstract])) OR (Acute Kidney Failures[Title/Abstract])) OR (Kidney Failures, Acute[Title/Abstract])) OR (Acute Kidney Failure[Title/Abstract])) OR (Acute Renal Failure[Title/Abstract])) OR (Acute Renal Failures[Title/Abstract])) OR (Renal Failures, Acute[Title/Abstract])) ) OR (Renal Failure, Acute[Title/Abstract])) OR (Renal Insufficiency, Acute[Title/Abstract])) OR (Acute Renal Insufficiencies[Title/Abstract])) OR (Renal Insufficiencies, Acute[Title/Abstract])) OR (Acute Kidney Insufficiency[Title/Abstract])) OR (Acute Renal Insufficiency[Title/Abstract])) OR (Kidney Insufficiency, Acute[Title/Abstract])) OR (Acute Kidney Insufficiencies[Title/Abstract])) OR (Kidney Insufficiencies, Acute[Title/Abstract])))) AND (("Risk Factors"[Mesh]) OR (((((((((((((((((((Risk Factors[Title/Abstract]) OR (Factor, Risk[Title/Abstract])) OR (Risk Factor[Title/Abstract])) OR (Population at Risk[Title/Abstract])) OR (Populations at Risk[Title/Abstract])) OR (Risk Scores[Title/Abstract])) OR (Risk Score[Title/Abstract])) OR (Score, Risk[Title/Abstract])) OR (Risk Factor Scores[Title/Abstract])) OR (Risk Factor Score[Title/Abstract])) OR (Score, Risk Factor[Title/Abstract])) OR (Health Correlates[Title/Abstract])) OR (Correlates, Health[Title/Abstract])) OR (Social Risk Factors[Title/Abstract])) OR (Factor, Social Risk[Title/Abstract])) OR (Factors, Social Risk[Title/Abstract])) OR (Risk Factor, Social[Title/Abstract])) OR (Risk Factors, Social[Title/Abstract])) OR (Social Risk Factor[Title/Abstract])))


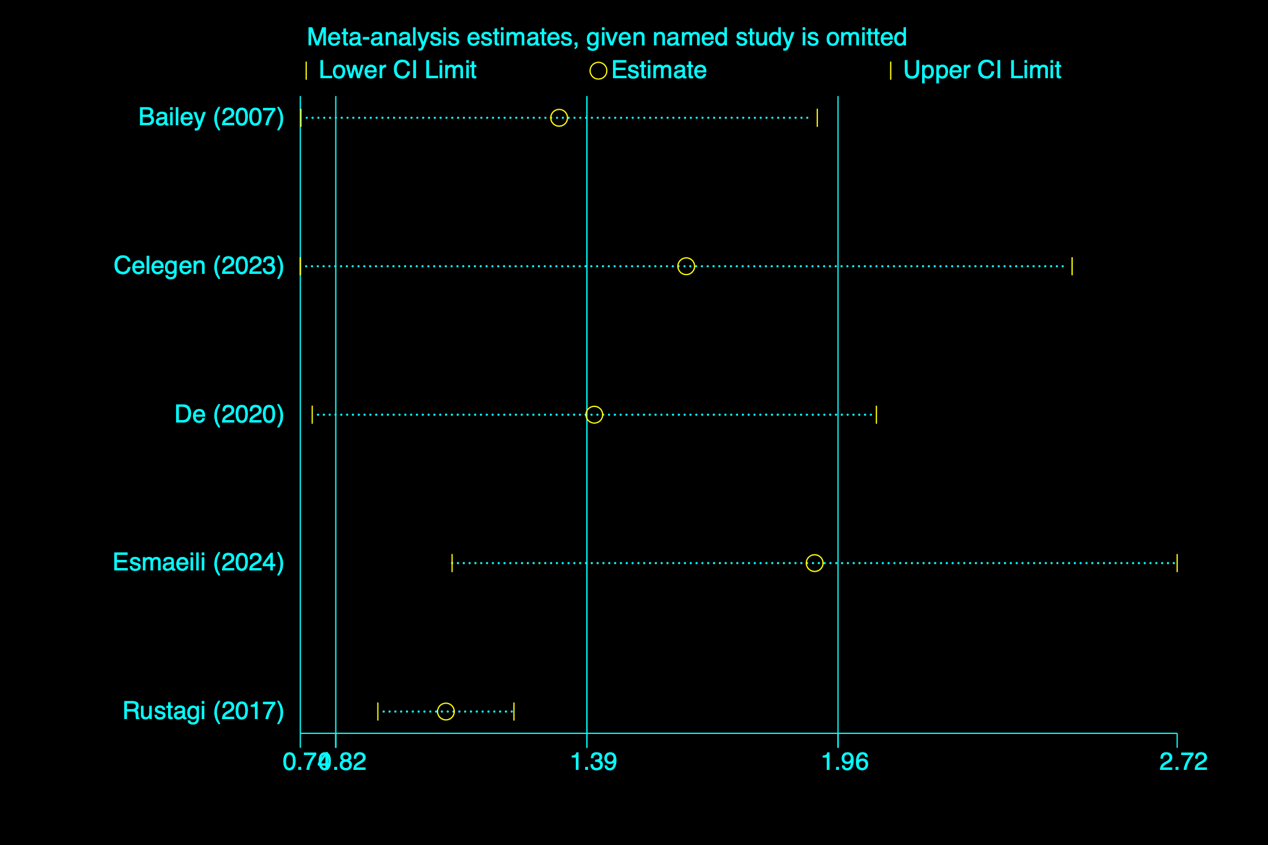


Figure S1 Results of sensitivity analysis of age (per year increase)


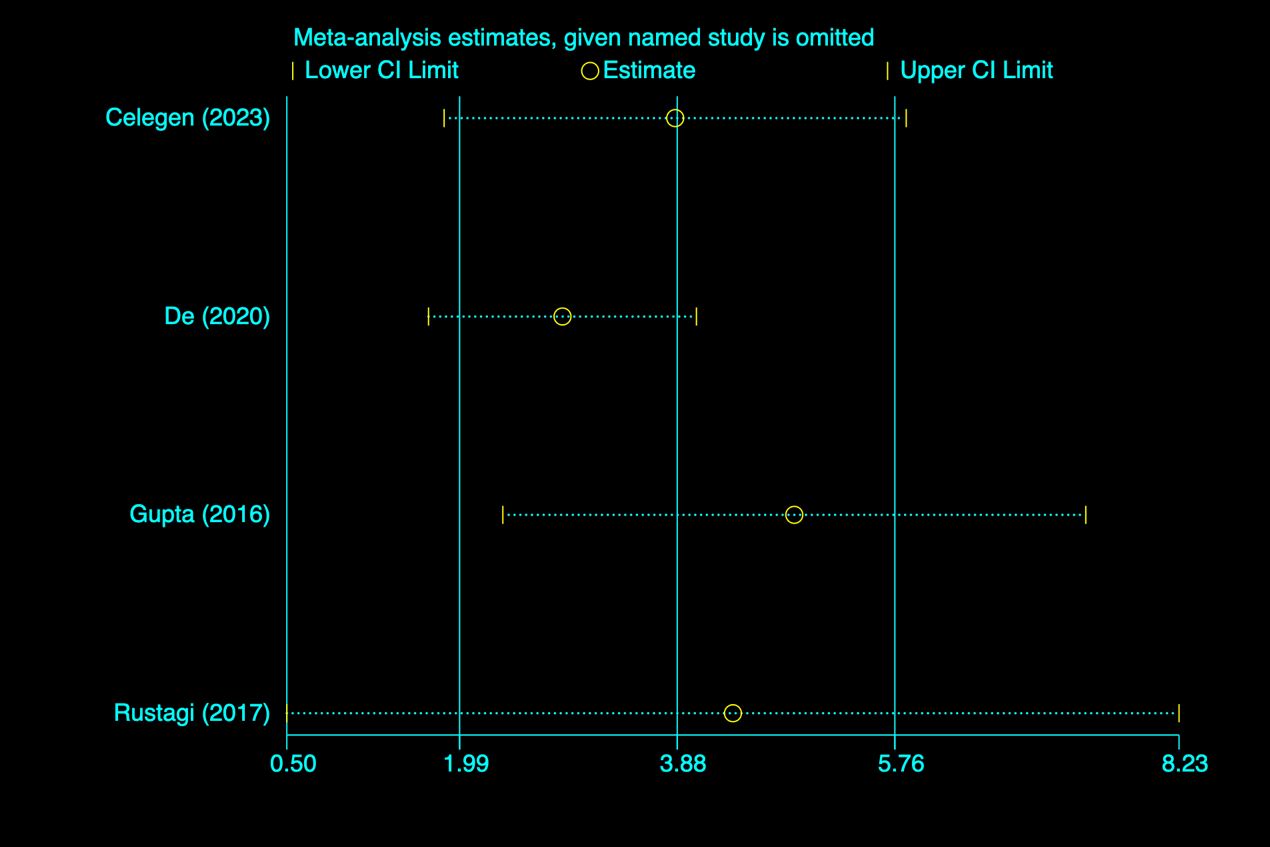


Figure S2 Results of sensitivity analysis of young multiple organ dysfunction syndrome


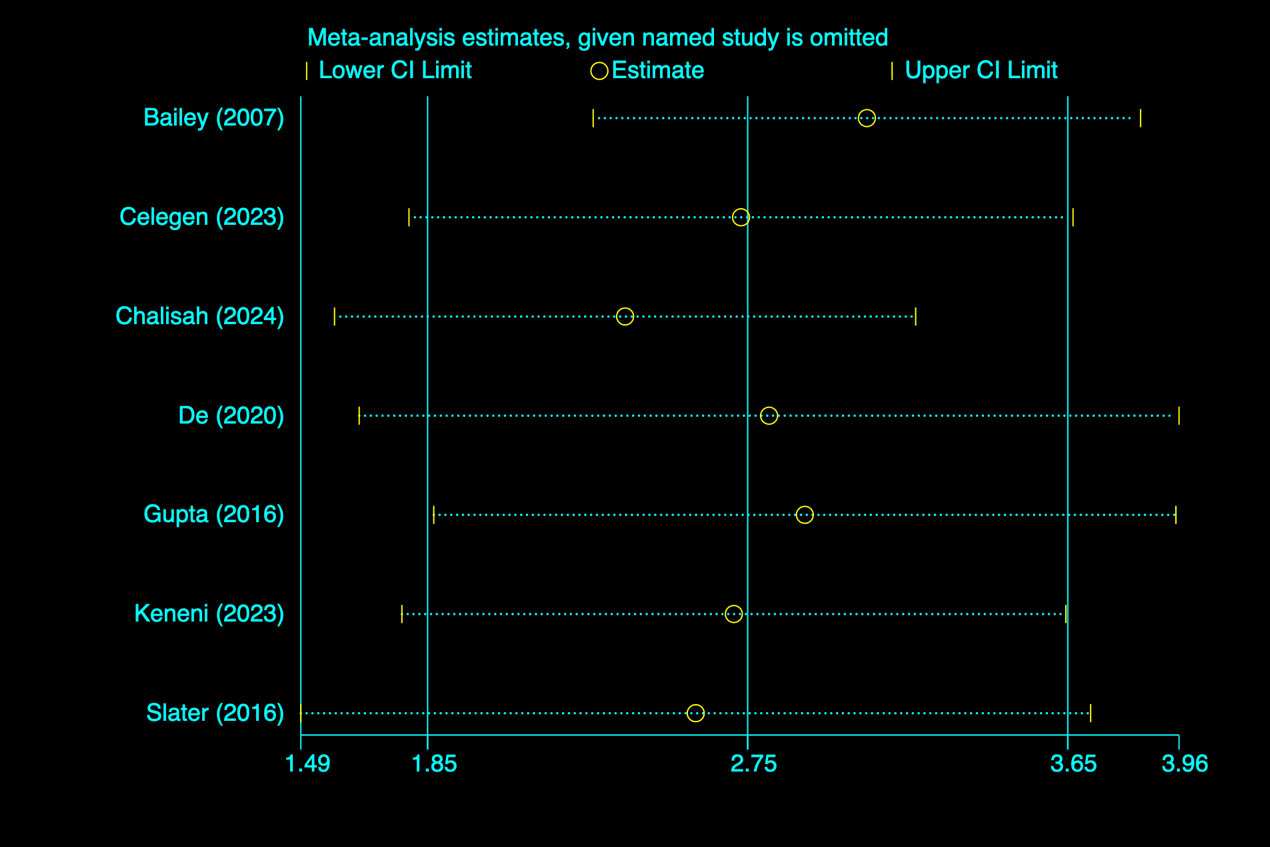


Figure S3 Results of sensitivity analysis of nephrotoxic drugs


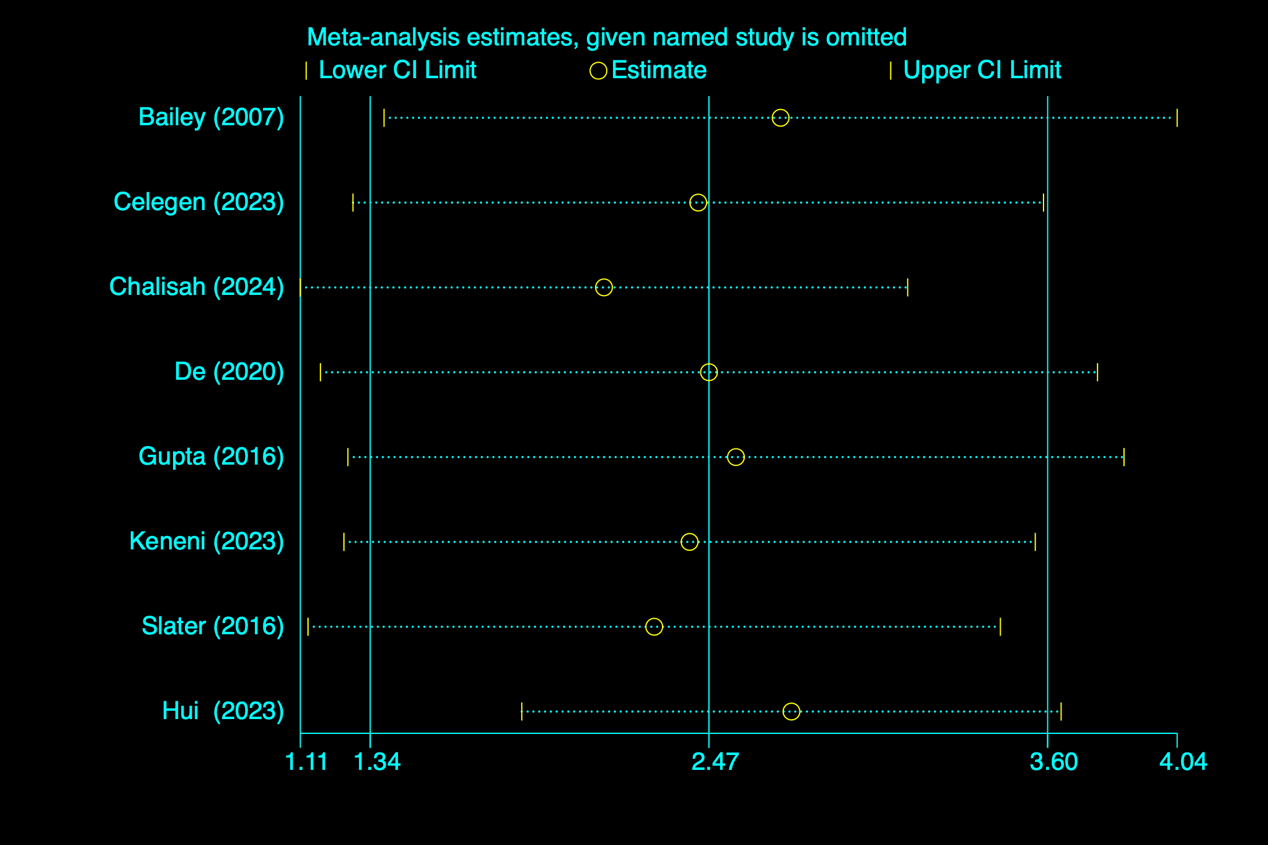


Figure S4 Results of sensitivity analysis of mechanical ventilation


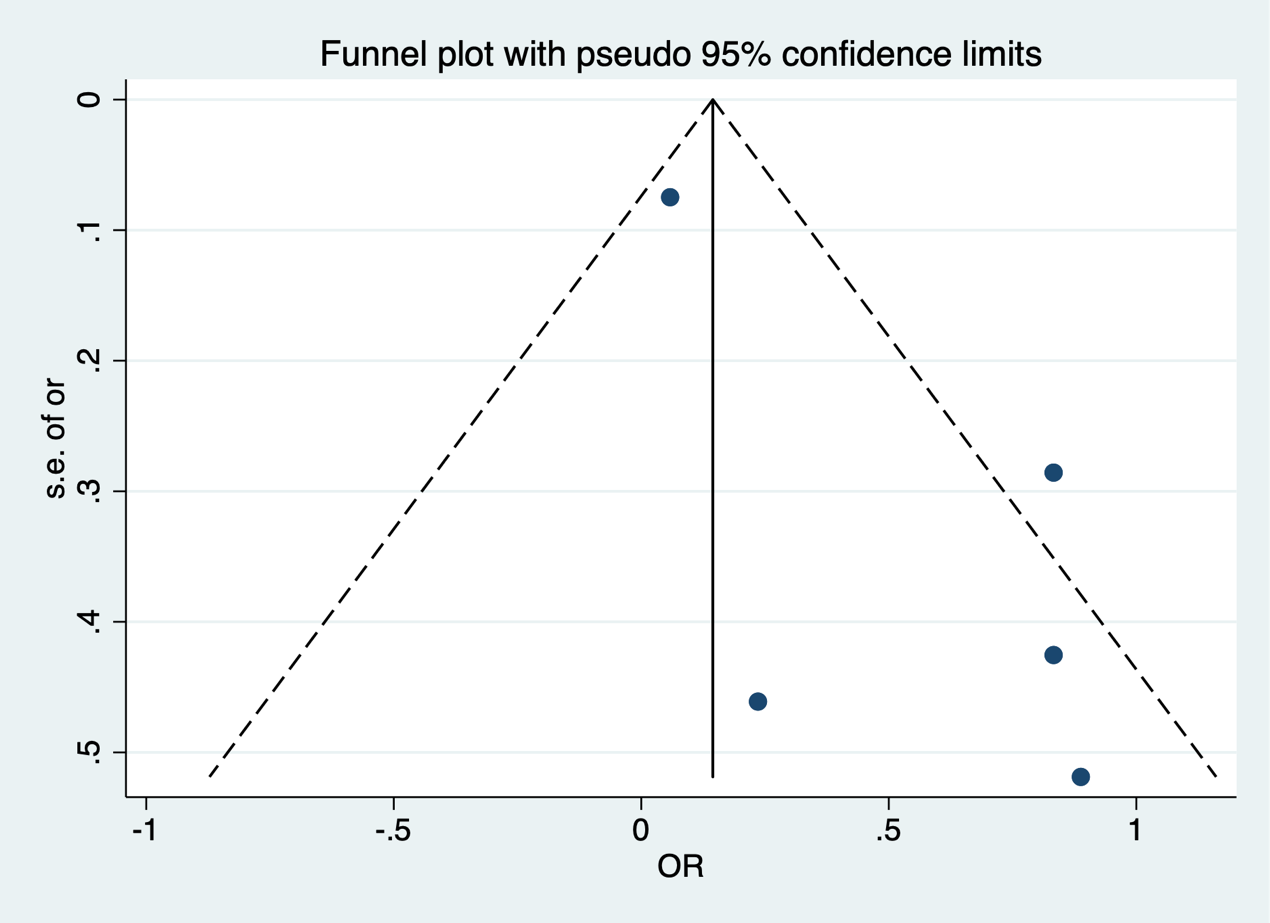


Figure S5 Funnel plot of meta-analysis of age (per year increase)


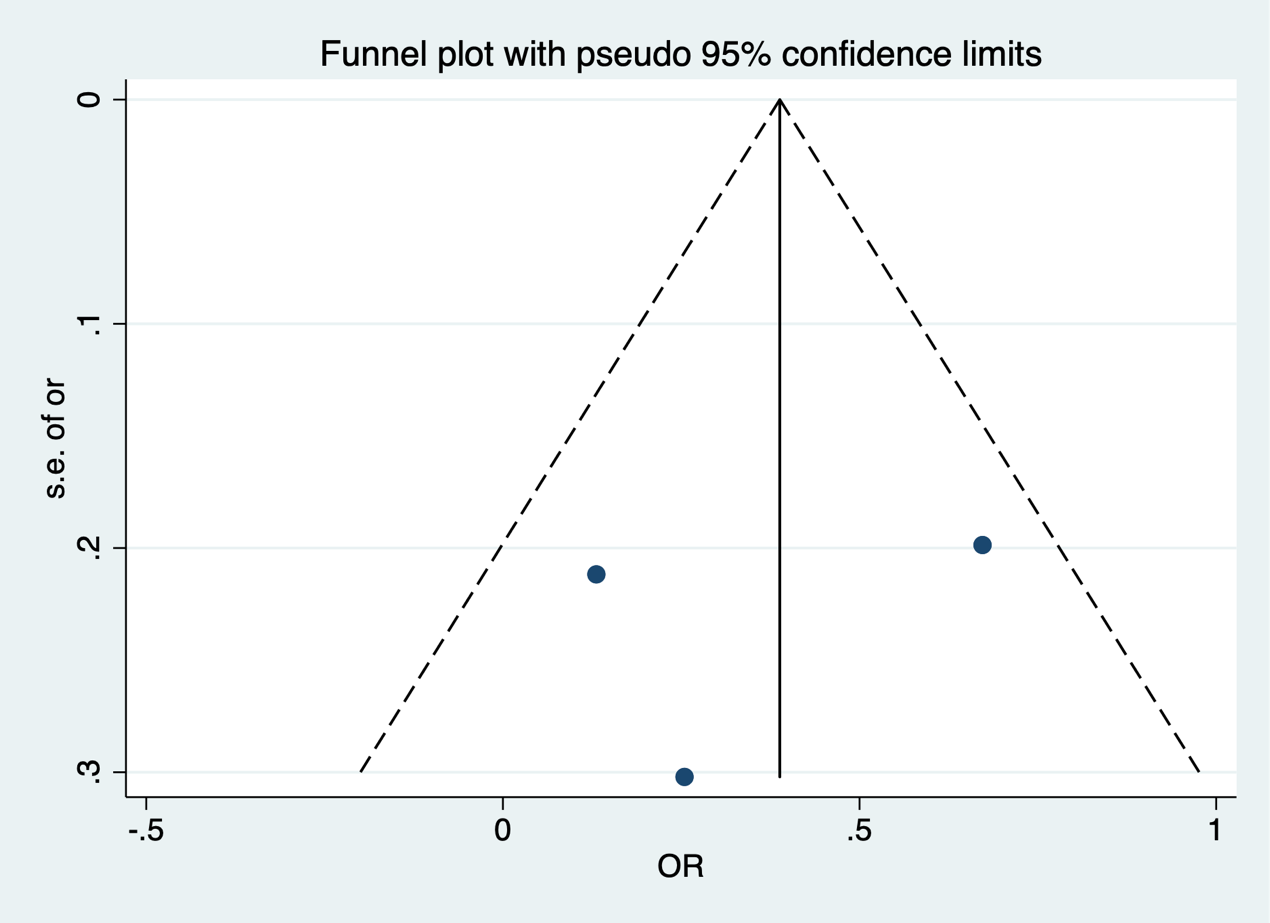


Figure S6 Funnel plot of meta-analysis of female


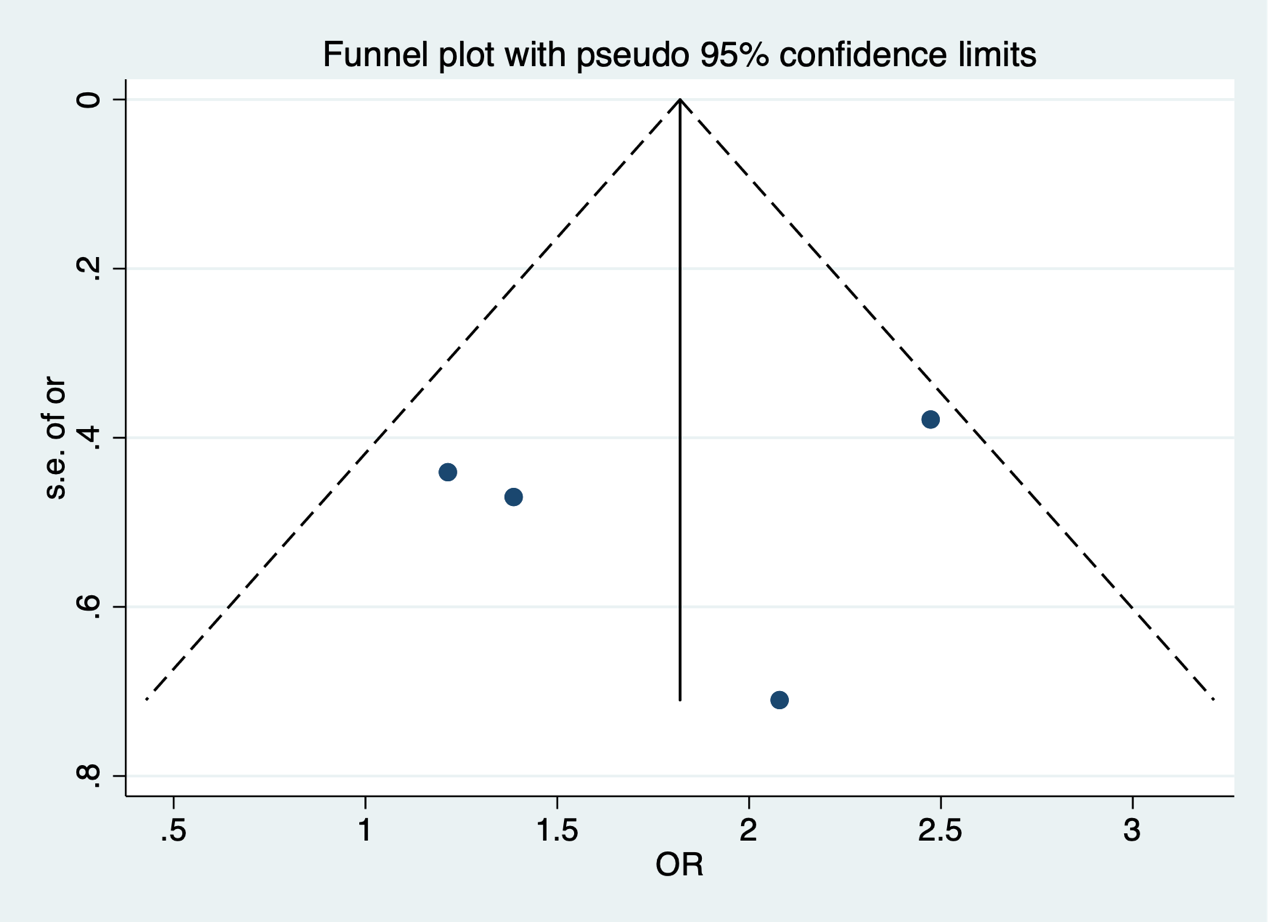


Figure S7 Funnel plot of meta-analysis of sepsis


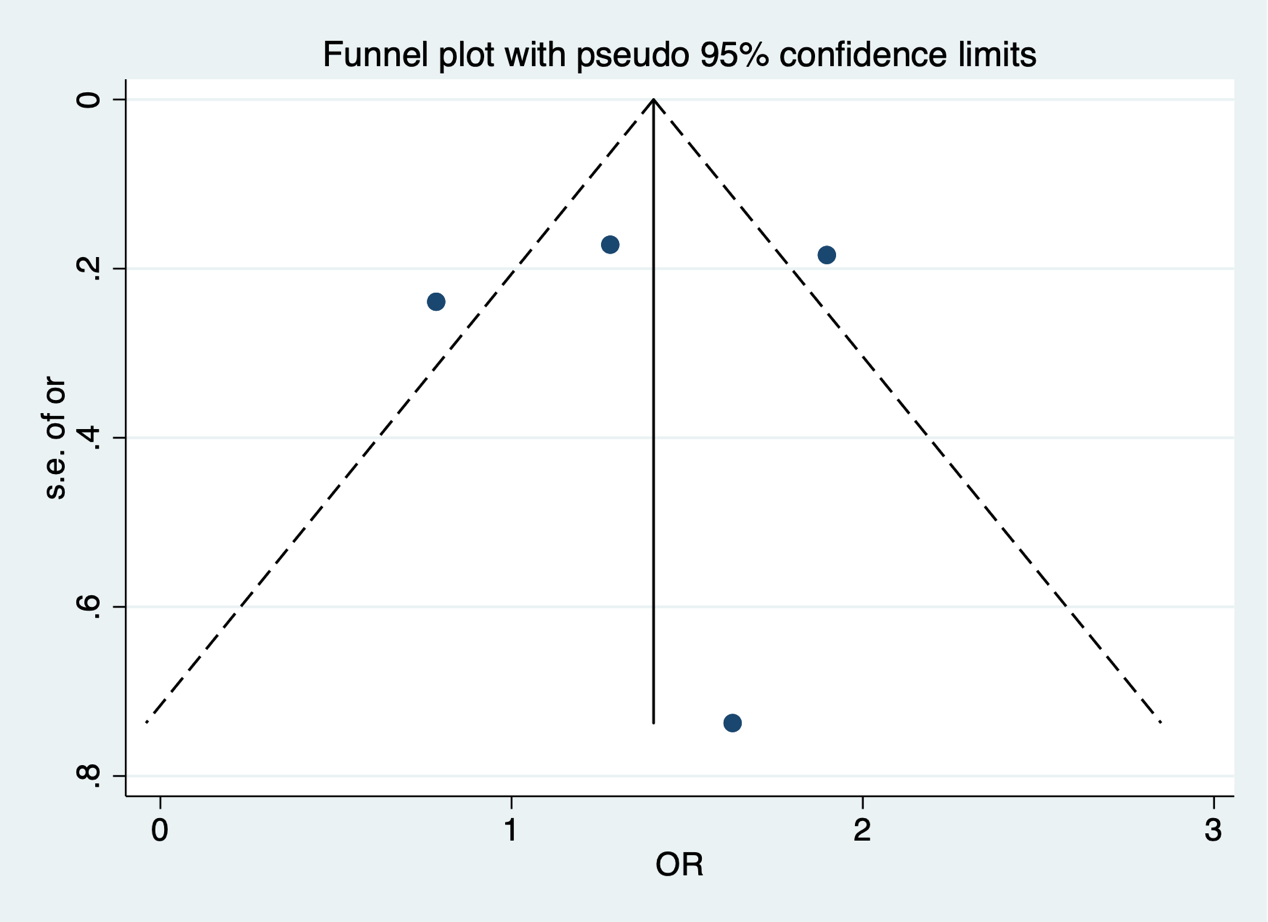


Figure S8 Funnel plot of meta-analysis of multiple organ dysfunction syndrome


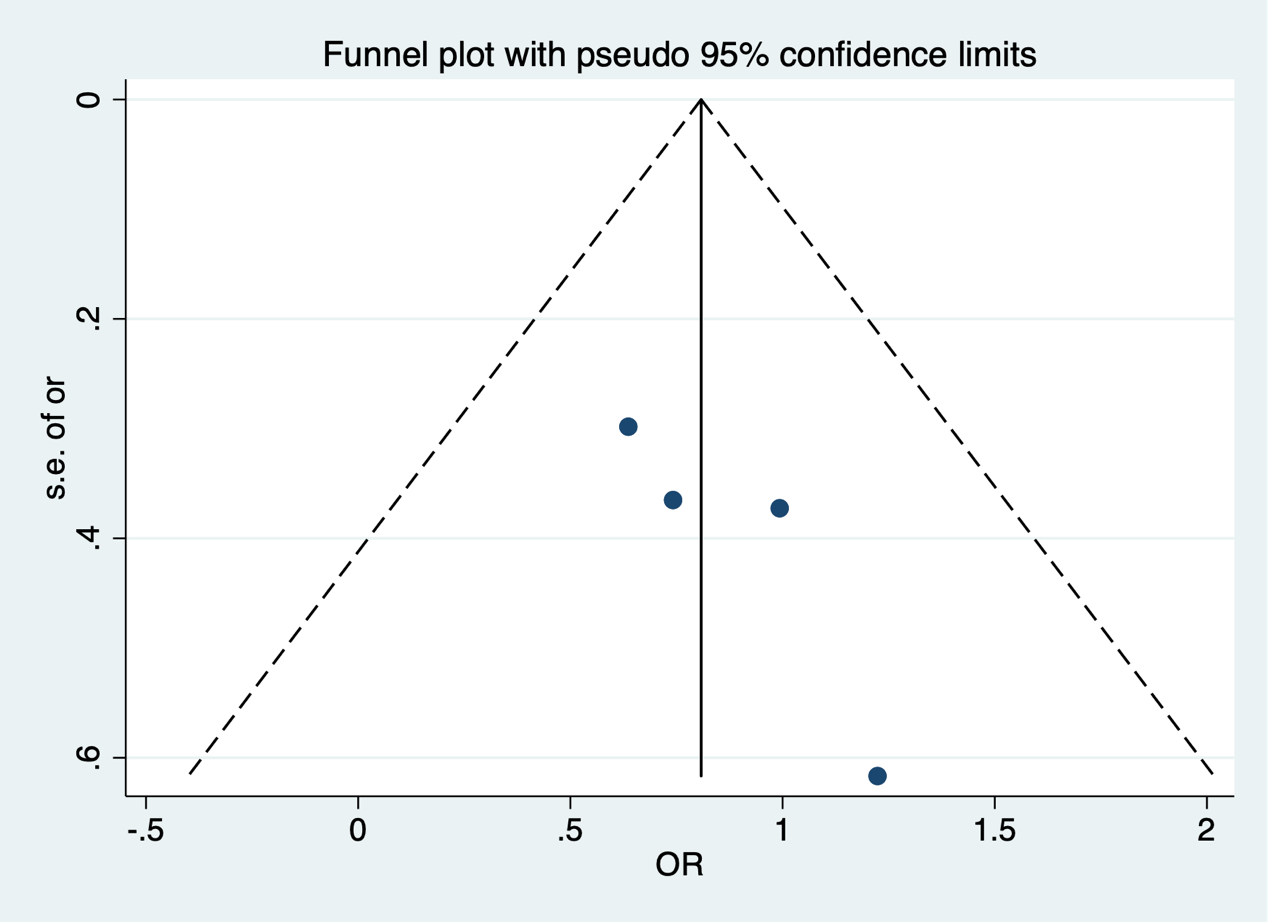


Figure S9 Funnel plot of meta-analysis of coagulopathy


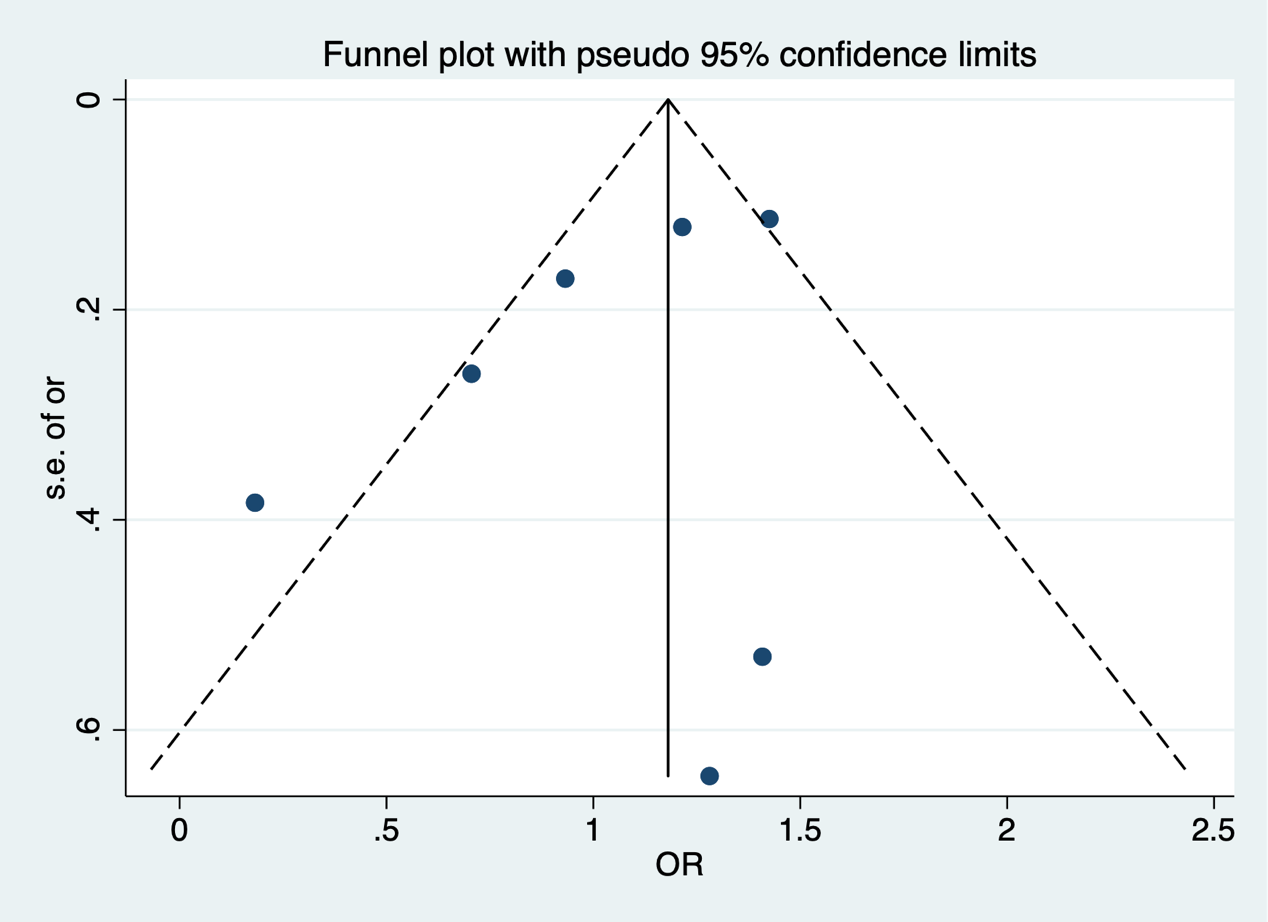


Figure S10 Funnel plot of meta-analysis of nephrotoxic drugs


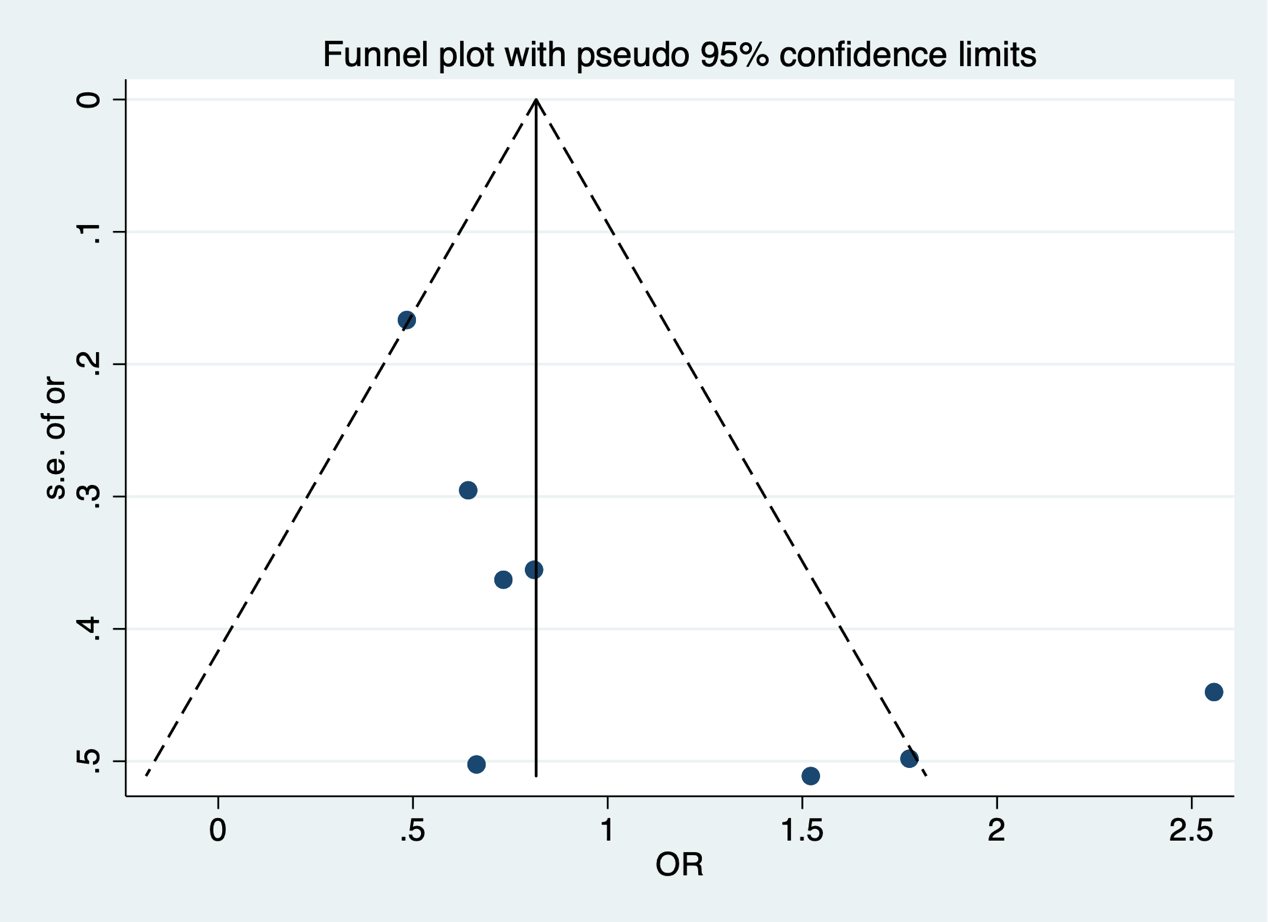


Figure S11 Funnel plot of meta-analysis of mechanical ventilation


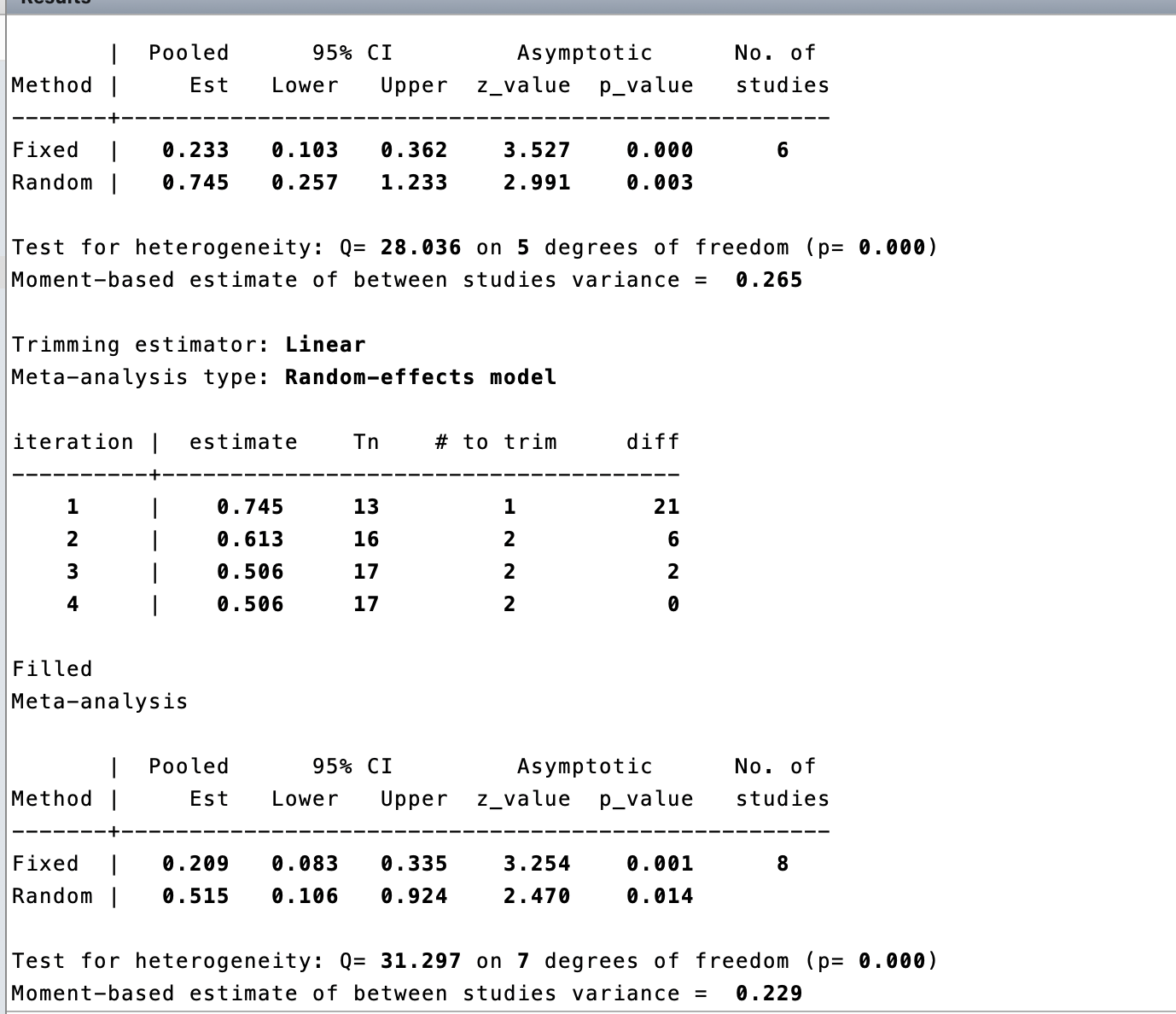


Figure S12 trim-and-fill results for young age


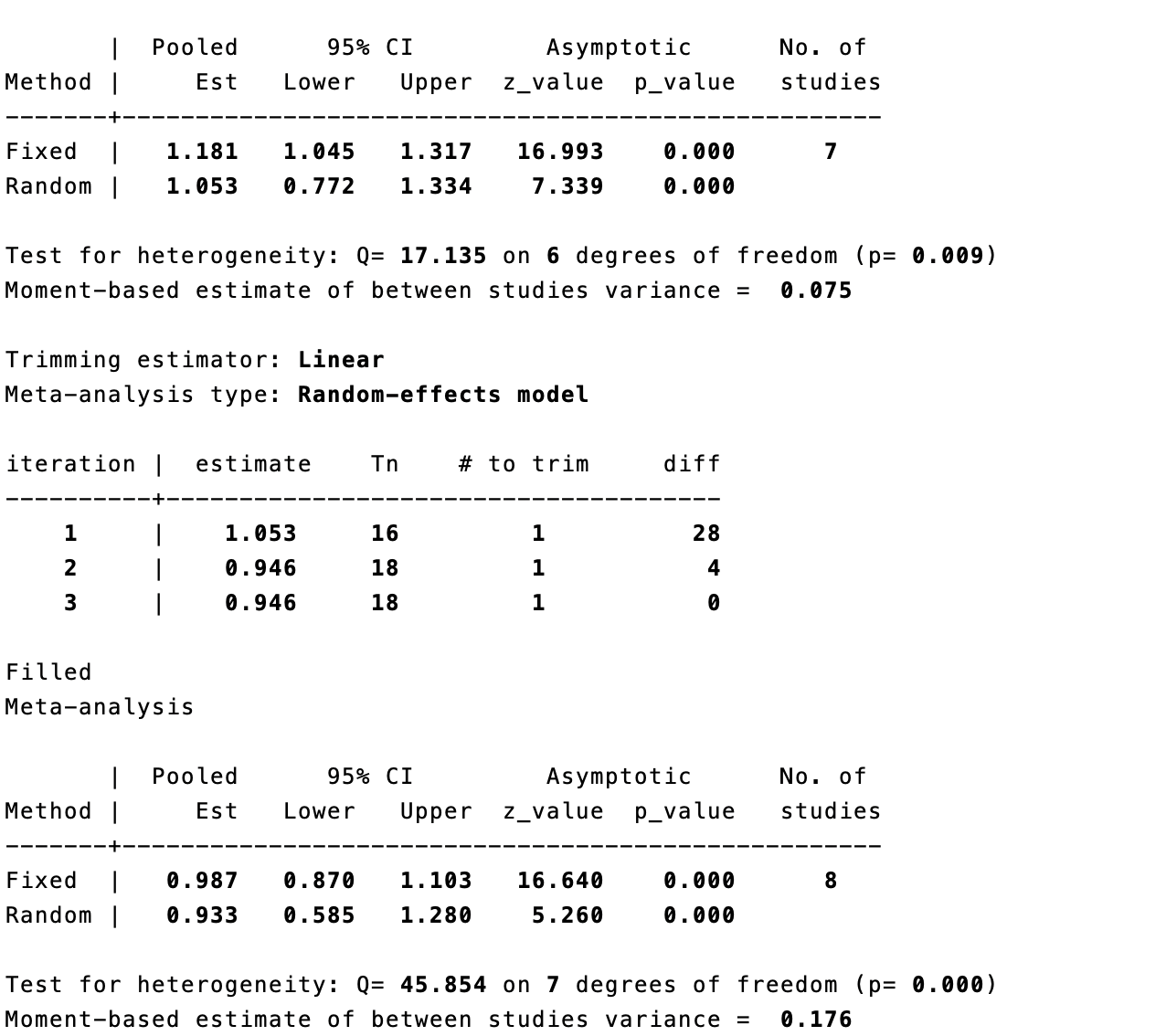


Figure S13 trim-and-fill results for nephrotoxic drugs


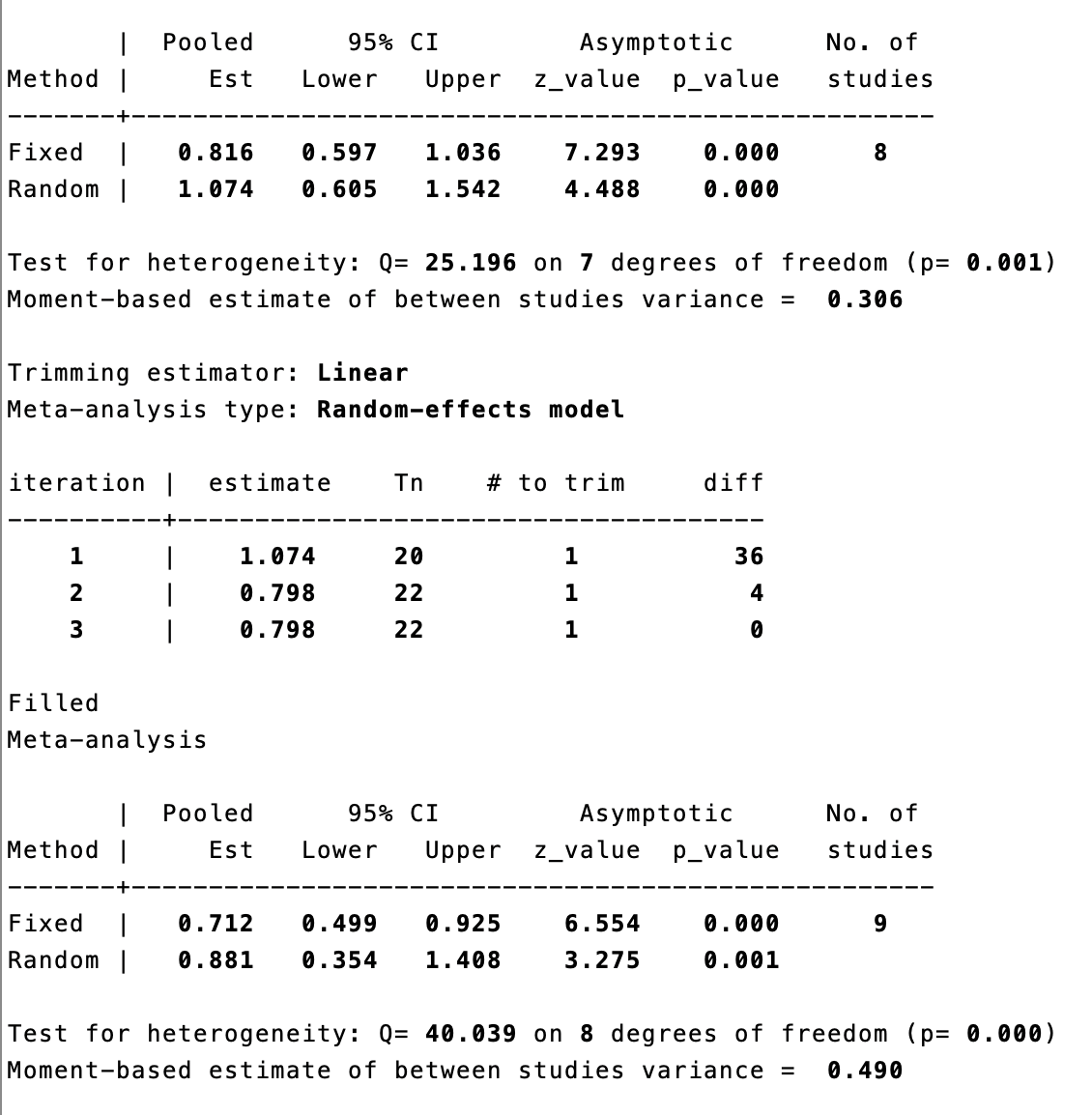


Figure S14 trim-and-fill results for mechanical ventilation
